# A New Hope for CD56^neg^CD16^pos^ NK Cells as Unconventional Cytotoxic Mediators: An Adaptation to Chronic Diseases

**DOI:** 10.3389/fcimb.2020.00162

**Published:** 2020-04-21

**Authors:** Catherine S. Forconi, Cliff I. Oduor, Peter O. Oluoch, John M. Ong'echa, Christian Münz, Jeffrey A. Bailey, Ann M. Moormann

**Affiliations:** ^1^Division of Infectious Diseases, Department of Medicine, University of Massachusetts, Worcester, MA, United States; ^2^Department of Pathology and Laboratory Medicine, Warren Alpert Medical School, Brown University, Providence, RI, United States; ^3^Center for Global Health Research, Kenya Medical Research Institute, Kisumu, Kenya; ^4^Laboratory of Viral Immunology, Experimental Immunology Institute, University of Zurich, Zurich, Switzerland

**Keywords:** natural killer cells, CD56^neg^CD16^pos^ subset, endemic Burkitt lymphoma, malaria, epstein-barr virus, transcription profile

## Abstract

Natural Killer (NK) cells play an essential role in antiviral and anti-tumoral immune responses. In peripheral blood, NK cells are commonly classified into two major subsets: CD56^bright^CD16^neg^ and CD56^dim^CD16^pos^ despite the characterization of a CD56^neg^CD16^pos^ subset 25 years ago. Since then, several studies have described the prevalence of an CD56^neg^CD16^pos^ NK cell subset in viral non-controllers as the basis for their NK cell dysfunction. However, the mechanistic basis for their cytotoxic impairment is unclear. Recently, using a strict flow cytometry gating strategy to exclude monocytes, we reported an accumulation of CD56^neg^CD16^pos^ NK cells in *Plasmodium falciparum* malaria-exposed children and pediatric cancer patients diagnosed with endemic Burkitt lymphoma (eBL). Here, we use live-sorted cells, histological staining, bulk RNA-sequencing and flow cytometry to confirm that this CD56^neg^CD16^pos^ NK cell subset has the same morphological features as the other NK cell subsets and a similar transcriptional profile compared to CD56^dim^CD16^pos^ NK cells with only 120 genes differentially expressed (fold change of 1.5, *p* < 0.01 and FDR<0.05) out of 9235 transcripts. CD56^neg^CD16^pos^ NK cells have a distinct profile with significantly higher expression of *MPEG1* (perforin 2), *FCGR3B* (CD16b), *FCGR2A*, and *FCGR2B* (CD32A and B) as well as *CD6, CD84, HLA-DR, LILRB1/2*, and *PDCD1* (PD-1), whereas Interleukin 18 (IL18) receptor genes (*IL18RAP* and *IL18R1*), cytotoxic genes such as *KLRF1* (NKp80) and *NCR1* (NKp46), and inhibitory *HAVCR2* (TIM-3) are significantly down-regulated compared to CD56^dim^CD16^pos^ NK cells. Together, these data confirm that CD56^neg^CD16^pos^ cells are legitimate NK cells, yet their transcriptional and protein expression profiles suggest their cytotoxic potential is mediated by pathways reliant on antibodies such as antibody-dependent cell cytotoxicity (ADCC), antibody-dependent respiratory burst (ADRB), and enhanced by complement receptor 3 (CR3) and FAS/FASL interaction. Our findings support the premise that chronic diseases induce NK cell modifications that circumvent proinflammatory mediators involved in direct cytotoxicity. Therefore, individuals with such altered NK cell profiles may respond differently to NK-mediated immunotherapies, infections or vaccines depending on which cytotoxic mechanisms are being engaged.

## Introduction

Natural Killer (NK) cells are crucial mediators of innate immune responses against virally infected and malignant cells (Herberman et al., 1975; Kiessling et al., 1975; Trinchieri and Santoli, 1978). NK cell function depends on a balance between activation and inhibition signals triggered by multiple surface receptors engaged during their surveillance of host cells (Long et al., 2013). NK cells were originally defined as CD3^neg^CD56^pos^ cells and represent 10 to 15% of lymphocytes in peripheral blood (Robertson and Ritz, 1990). CD56 is a Neural Cell Adhesion Molecule 1 (NCAM-1) involved in cell-to-cell and cell-to-matrix interactions (Lanier et al., 1991) and its expression varies with NK cell maturation. Of the peripheral NK cells, ~10% are CD56^bright^ NK cells which are essential for pro-inflammatory cytokine production (Cooper et al., 2001) particularly when they also express CD62L, an adhesion/homing molecule (Cichocki et al., 2016) and are less cytotoxic (Jacobs et al., 2001); whereas CD56^dim^ NK cells comprise ~90% of NK cells in healthy adults and have low cytokine production but high cytotoxic capacity (Cooper et al., 2001; Jacobs et al., 2001). CD56^dim^ NK cells are polyfunctional and play either an immunoregulatory role as canonical CD56^dim^ NK cells characterized as CD62L^neg^CD57^pos^ Eomesodermin^pos^ (Eomes) Promyelocytic Leukemia Zinc Finger^pos^ (PLZF) (Cichocki et al., 2016) or are considered adaptive NK cells which do not express PLZF or FcRγ (also referred to as an Immunoreceptor Tyrosine-based Activation Motif (ITAM)-bearing transmembrane adapter protein). Adaptive NK cells are involved in immunosurveillance with induction of cytotoxic granules (perforin and granzymes) upon engagement with CD16, NKG2C or activating Killer Immunoglobulin-like Receptor (KIR) (Hwang et al., 2012; Schlums et al., 2015; Tesi et al., 2016). Recently, adaptive CD56^dim^ NK cells were associated with protection from *Plasmodium falciparum* (*Pf* ) malaria (Hart et al., 2019). *Pf-*exposed individual had a higher frequency of FcRγ^neg^ adaptive CD56^dim^ NK cells and displayed increased antibody-dependent cellular cytotoxicity (ADCC) against *Pf-*infected red blood cells (Hart et al., 2019).

CD56^neg^CD16^pos^ NK cells were discovered 25 years ago in Human Immunodeficiency Virus (HIV) patients (Hu et al., 1995) and has been shown to expand during other chronic infections, such as Hepatitis C Virus (HCV), especially those who failed treatment (Mavilio et al., 2003, 2005; Gonzalez et al., 2009; Prada et al., 2013) and more recently in human Cytomegalovirus (HCMV) and Epstein-Barr virus (EBV) co-infected elderly individuals (>60 years of age) (Müller-Durovic et al., 2019). Compared to CD56^pos^ NK cells, CD56^neg^ NK cells have been portrayed as “dysfunctional” because of lower expression of cytotoxic receptors such as NKp46 and NKp30, low cytokine production as well as reduction of natural cytotoxicity (Mavilio et al., 2005; Müller-Durovic et al., 2019). More recently, we reported a dramatic expansion of CD56^neg^CD16^pos^ NK cells in African children chronically/repeatedly infected with *Plasmodium falciparum* malaria and in those who were diagnosed with endemic Burkitt lymphoma (eBL) (Forconi et al., 2018). Proteomic analyses showed similarities between CD56^dim^CD16^pos^ and CD56^neg^CD16^pos^ NK cells (Voigt et al., 2018) thus supporting the classification of this subset as NK cells. Since CD56^neg^CD16^pos^ NK cells are extremely low in American/European healthy adults (Supplemental Figure 1), most studies have focused on characterizing the function and therapeutic potential of CD56^bright^ and CD56^dim^ NK cell subsets. However, it appears that healthy adults from western Kenya also have a significant proportion of CD56^neg^CD16^pos^ NK cells, similar to children chronically/repeatedly infected with *Pf-*malaria (Supplemental Figure 1). Besides the emerging evidence associating this NK cell subset with chronic infections, the development and function of CD56^neg^CD16^pos^ NK cells have only begun to be explored.

Endemic BL is an Epstein-Barr virus (EBV) associated, aggressive pediatric cancer that occurs in regions of equatorial Africa with high *P. falciparum* transmission, i.e., holoendemic malaria (Burkitt, 1962). EBV is a herpesvirus which has evolved to evade immune clearance in order to establish a life-long, asymptomatic infection within immunocompetent individuals (Schmiedel and Mandelboim, 2017). Children residing in malaria holoendemic areas, where eBL incidence is high, are usually infected by EBV before 2 years of age (Piriou et al., 2012). At the same time these children are repeatedly infected with *P. falciparum* which in turn induces episodes of viral reactivation resulting in higher EBV loads (Moormann et al., 2005; Piriou et al., 2012; Reynaldi et al., 2015). *P. falciparum* malaria is postulated to diminish EBV-specific immune surveillance as a component of eBL etiology, a cancer common in children aged 5–9 years (Moss et al., 1983; Whittle et al., 1984; Moormann et al., 2007, 2009; Njie et al., 2009; Snider et al., 2012; Chattopadhyay et al., 2013; Parsons et al., 2016). NK cells have been independently shown to help control both of these infections, killing EBV-infected B cells during adolescent acute infectious mononucleosis (AIM) (Azzi et al., 2014) and malaria-infected red blood cells (Horowitz et al., 2010; Wolf et al., 2017). However, little is known about NK cell function during EBV and malaria co-infections and their role in protection against eBL pathogenesis.

In order to further clarify similarities and differences between CD56^dim^CD16^pos^ and CD56^neg^CD16^pos^ NK cells we performed histology staining, bulk RNA sequencing and protein expression profile validation by flow cytometry using fluorescence-activated cell sorting (FACS) of NK subsets of peripheral blood mononuclear cells (PBMCs) isolated from children who had life-long exposure to *P. falciparum* infections and were diagnosed with eBL.

## Methods

### Study Population and Ethical Approvals

Ethical approval was obtained from the Scientific and Ethics Review Unit (SERU) at the Kenya Medical Research Institute (KEMRI) and the Institutional Review Board at the University of Massachusetts Medical School, Worcester, USA. Written informed consent was obtained from adults and from parents of minor study participants. Healthy children and adults were recruited at a rural health center in Kenya. Inclusion criteria for children were EBV sero-positivity, HIV-negative and born to HIV-negative mothers. Inclusion criteria for Kenyan and American adults was HIV-negative status. Children with suspected eBL were enrolled at Jaramogi Oginga Odinga Teaching and Referral Hospital (JOOTRH) in Kisumu, Kenya. Two independent pathologists confirmed diagnosis by cyto-pathology and May-Grunwald Giemsa staining. Tumor samples were further characterized by transcriptome and mutational profiling to confirm eBL diagnosis (Kaymaz et al., 2017). This cancer is more prevalent in male compared to female children, with a peak-age incidence ranging from 5 to 9 years old (Buckle et al., 2016), and at the time of this study, we only had sufficient samples from male eBL children. Therefore, baseline peripheral blood samples were used from 8 male eBL children before induction of chemotherapy. However, we have previously shown that both male and female eBL patients have significantly elevated frequencies of CD56^neg^CD16^pos^ NK cells (Forconi et al., 2018).

### ddPCR to Quantify EBV Load

For each patient, 500 μl of blood was collected in an EDTA microtainer tube. After 5 min of centrifugation, 200 μl of plasma was separated from the blood cell pellet and replaced by an equivalent amount of 1X PBS, pH 7.4. Using the whole blood DNA extraction kit from Qiagen, DNA was isolated from the PBS resuspended blood cell pellet and total DNA concentration was measured by NanoDrop (Thermo Fisher Scientific). We used digital droplet PCR (ddPCR) to determine EBV load in each sample by amplifying EBV BALF5 and human β-actin gene, using primers and probes shown in Table 1. The duplex ddPCR reactions were prepared in a total volume of 20 μl which contained 10 μl of ddPCR Supermix for probes (No UTP) (Bio-Rad Laboratories), and 2 sets of each primer and probe combination (0.9 μM of primers and 0.25 μM of probes). The BioRad Automated Droplet Generator (AutoDG) (Bio-Rad Laboratories) was used to ensure consistent droplet generation. After the ddPCR reaction, the number of positive and negative droplets were counted by the Bio-Rad QX200TM Droplet reader and EBV viral loads quantified as copies/ng human DNA.

**Table 1:** Droplet Digital PCR (ddPCR) EBV and Human Primer and Probe Sequences.

	**Sequence 5^**′**^-3^**′**^**
EBV-BALF5 FP	GAAGCCCTCTGGACTTCCATG
EBV-BALF5 RP	CCCTGTTTATCCGATGGAATG
EBV-BALF5 Probe	FAM -TGTACACGCACGAGAAATGCGCCT-BHQ-1
Human β-actin FP	GCTCATGGCAAGAAAGTGCTC
Human β-actin RP	GCAAAGGTGCCCTTGAGGT
Human β-actin Probe	HEX-AGTGATGGCCTGGCTCACCTGGAC-BHQ

*FP, Forward primer; RP, Reverse primer*.

### Multiplex Suspension Bead-Based Serology Assay

To measure IgG antibody levels in the plasma fraction, we used a Luminex bead-based suspension assay as previously published (Cham et al., 2009; Forconi et al., 2018). In brief, antibodies to Viral Capsid Antigen (VCA) and Epstein-Barr nuclear antigen 1 (EBNA1) (gift from Jaap Middeldorp, Cyto-Barr) were used to determine EBV seropositivity (Middeldorp and Herbrink, 1988; van Grunsven et al., 1994). Previous *P. falciparum* exposure was determined using recombinant proteins to blood stage malaria antigens: apical membrane antigen 1 (AMA1) and merozoite surface protein (MSP1) (gifts from Sheetji Dutta, Evelina Angov, and Elke Bergmann from the Walter Reed Army Institute of Research). Briefly, 200 μg of each antigen or bovine serum albumin (BSA) (Sigma-Aldrich) were coupled to carboxylated beads microspheres (BioRad) and then incubated with plasma samples from our study participants, followed by incubation with biotinylated anti-human IgG diluted 1:1000 and streptavidin (BD Pharmingen) diluted 1:1000 following manufacturer's instructions. Antigen-specific fluorescence values were quantified on a BioPlex 200 multi-analyte analyzer with subtraction of fluorescence values obtained with BSA-conjugated beads for each patient. Results are reported as median fluorescence intensity (MFI) after acquisition of a minimum of 50 beads per antigen.

### Fluorescence-Activated Cell Sorting (FACS) of NK Subsets

**Figure 1:**
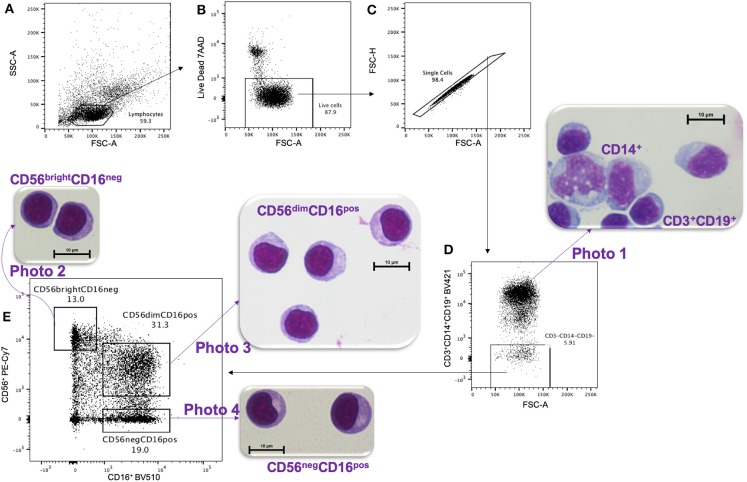
Gating strategy for FACS and HandE staining. PMBCs were gated on **(A)** lymphocytes size determined by SSC-A vs. FSC-A cytoplots, then **(B)** live, and **(C)** single cells were selected. **(D)** A Dump channel was used to eliminate CD3^+^, CD14^+^, and CD19^+^ cells. These cells were stained purple using hematoxylin and eosin (HandE), as shown in **photo 1**. Then **(D)** CD3^−^CD14^−^CD19^−^ cells were **(E)** sorted based on CD56 vs. CD16 expression and the three isolated NK cell subsets were stained with HandE: **photo 2** CD56^bright^CD16^neg^, **photo 3** CD56^dim^CD16^pos^, and **photo 4** CD56^neg^CD16^pos^.

Peripheral blood mononuclear cells (PBMCs) were isolated by Ficoll-Paque density gradient centrifugation and cryopreserved until use (viability > 97%). PBMCs were thawed at 37°C in complete media composed of RPMI, 10% heat-inactivated Fetal Bovine Serum (MilliporeSigma), 2 mM L-glutamine, 1X Penicillin/Streptomycin and 10 mM HEPES (Invitrogen). NK cell subsets were then isolated using a 16-color BD FACS 2-ARIA II cell sorter at the UMASS Flow Cytometry Core. The gating strategy (Figure 1) used the following antibody/fluorochrome combinations identified by the Resource Identification Portal number RRID to isolate NK subsets: CD16-BV510 (RRID: AB_2562085) and CD56-PECy7 (RRID: AB_399970) with the exclusion of dead cells by 7'AAD (BD Pharmingen, Cat#559925) and dump channel for cells expressing either CD3-BV421 (RRID: AB_10962690), CD14-BV421 (RRID: AB_2563296), or CD19-BV421 (RRID: AB_11142678). In order to compare cell morphology between CD56^dim^CD16^pos^ and CD56^neg^CD16^pos^ NK cell subsets, we also collected the CD56^bright^CD16^neg^ NK cells and CD3^+^CD14^+^CD19^+^ cells.

### Morphology by Light Microscopy

To examine morphology, live-sorted cells from each subset were cytospun on a slide and stained with hematoxylin and eosin (H&E) following Hema 3™ Stat Pack kit instructions (Fisherbrand Cat#122-911). Hematoxylin stains the nucleus deep blue-violet whereas eosin stains the elastin/collagen/reticular fibers of the cell pink. Slides were imaged on Nikon microscope Eclipse E400 with ocular lens 10x and 100x objectives using the DS-Qi1MC and Digital Sight DS-U3 (Nikon) camera system and NIS-Element BR version 4.20 software. Images were analyzed by a clinical pathologist and transfusion specialist at UMass Medical School to determine cell types.

### RNA Sequencing

Live-FACS sorted NK cell subsets (CD56^dim^CD16^pos^ and CD56^neg^CD16^pos^) were immediately collected in 4°C 2 × Buffer TCL with (2%) β-mercaptoethanol (Qiagen). Total RNA was isolated and strand-specific ribosomal RNA-depleted sequencing libraries were generated using the standard protocol of SMARTer Stranded Total RNA-Seqv2 Pico input kit (Takara Bio). Given the small quantity of total RNA that was obtained from the ~5,000 NK cells sorted for each subset, the depletion of abundant rRNA was performed after cDNA synthesis using probes specific to mammalian rRNA. Sequencing libraries were purified using XP Ampure magnetic beads (Beckman Coulter Inc.) after each reaction step. Final libraries were amplified using SeqAmp DNA polymerase, and qualities and concentrations were measured with a Bioanalyzer Agilent High sensitivity DNA kit. Samples were sequenced on an Illumina HiSeq 4000 (Illumina, Inc.), obtaining depths of 10–20 million paired-end 50 bp reads for each NK cell subset sequenced. Sequencing files were deposited in the NCBI's database of Genotypes and Phenotypes (dbGaP) with accession number phs001282.v2.p1.

### Differential Gene Expression Analysis

Differential gene expression was performed using standard methods. Sequence reads were first checked for quality using FastQC (Andrews, 2014) and sorted by sample based on the unique sample indexes identified by Novobarcode (Novocraft Technologies). Residual Illumina 3'-end adaptor sequences and template switching oligos introduced during the cDNA synthesis were trimmed using Cutadapt (Martin, 2011). Paired reads were then aligned to a transcriptome index built by RNA-Seq by expectation-maximization (RSEM) (Li and Dewey, 2011) using Gencode annotation version 19 for protein coding transcripts and hg19 genomic sequence. RSEM calculated strand-specific expected read counts for each gene and gene expression count matrices for each NK cell subset were generated for downstream statistical analyses that were performed with R software (https://www.R-project.org/) (version 3.5.3).

Differential gene expression analysis between NK cell subsets (CD56^dim^CD16^pos^ and CD56^neg^CD16^pos^) was performed using R package edgeR (Robinson et al., 2010) which implements a Trimmed mean of M-values (TMM) normalization and a negative binomial approach (Robinson and Oshlack, 2010). We removed from the analysis all genes for which all counts per million (CPM) values were lower than 5 cpm. To control multiple testing, we applied the Benjamini-Hochberg procedure (BH) with threshold for statistical significance set at an adjusted *p*-value < 0.01 and false discovery rate (FDR) < 0.05.

### Validation by Flow Cytometry

PBMCs from 3 eBL children (2 of them were also used for RNA-sequencing) and 3 healthy Kenyan children were thawed and FACS live-sorted, as described above. In addition, we included the following antibodies: CD62L-PerCP5.5 (RRID: AB_2239105), DNAM1-BV711 (RRID: AB_2738956), TIM3-BV650 (RRID: AB_2565829), PD-1-APC-Fire750 (RRID: AB_2616721) and granzyme B-APC (RRID: AB_1500190); CD32-PerCP5.5 (RRID: AB_2616924) and IL18Ra-APC (RRID: AB_2800828). Data was acquired on a 19-color BD LSRII at the UMASS FACS Core and analyzed using FlowJo v10.6.0, R v3.5.1 and Prism v8.3.0. Non-parametric two-tailed paired Wilcoxon *t*-test was used and a *p*-value < 0.05 was considered significant.

### gProfiler

The genes identified to be significantly differentially expressed (*p*-value < 0.01 and FDR < 0.05) between the NK cell subsets were further analyzed using the gProfiler software (https://biit.cs.ut.ee/gprofiler) (Raudvere et al., 2019) to explore the potential functional consequences and associated pathways. gProfiler was run using g:GOSt ordered query with Bonferroni correction and a threshold of 0.01 equating to a *p*-unadjusted <10.E-16.

## Results

### Characterization of the Samples

Initial samples at diagnosis prior to chemotherapy from 8 eBL children were used for this study with a median age of 9.5 years (Table 2). Serology for the two pathogens associated with eBL was assessed and all children were seropositive for both EBV and *P. falciparum*. Survival outcomes varied with five eBL children (62%) being long-term (>2 year) survivors and three (38%) in-hospital deaths. Half of the children had low EBV loads (median 0.5 EBV copies/ng of human DNA) and half had high EBV loads (median 23.3 copies/ng of human DNA). No child in our study had lymphopenia with a median absolute lymphocyte count (ALC) of 3.5 × 10^5^ lymphocytes per μl blood (Shapiro et al., 1998). Finally, the percentage of each NK subset was assessed within the total NK cell population. Consistent with our previous study (Forconi et al., 2018), eBL children had elevated CD56^neg^ CD16^pos^ NK cell subset, with a median of 30.3%.

**Table 2:** Patients characteristics.

	**eBL (*n* = 8)**
Age^†^:	9.5 [6.5–11.75]
Sex: (Male)	8
Hemoglobin (g/dl)^†^	10.80 [9.75–11.55]
ALC: (10^3^ lymphocytes per μl)^†^	346.8 [214.1–418.4]
Serology in Median Fluorescence Intensity (MFI):	8,848 [6,595–24,668]
EBNA1^†^	
VCA^†^	28,114 [15,487–31,459]
MSP-1 (3D7)^†^	27,785 [22,058–29,767]
AMA-1^†^	26302 [21,173–31,008]
Survivors:	5 (62%)
Non-survivors:	3 (38%)
Low EBV viral load: (eBL *n* = 4)^†^ EBV copies/ng human DNA	0.5 [0.11–0.7]
High EBV viral load: (eBL *n* = 4)^†^ EBV copies/ ng human DNA	23.35 [7.47–245.1]
Tumor localization	
Jaw:	3 (38%)
Abdomen:	5 (62%)
% CD56^dim^ within NK cells^†^:	56.6 [34.55–67.1]
% CD56^neg^ within NK cells^†^:	30.30 [24.23–57.1]

*Median [25–75% interquartile]*.

### Morphology of CD56^neg^CD16^pos^ Cells Similar to Other NK Subsets

After live-sorting, an aliquot of each cell subset was fixed and stained by H&E in order to assess their morphology (Figure 1). All three NK cell subsets CD56^bright^CD16^neg^ (Figure 1, photo 2), CD56^dim^CD16^pos^ (Figure 1, photo 3) and CD56^neg^CD16^pos^ (Figure 1, photo 4) had similar morphology by microscopy, round in shape, ~10 μm in size and with a prominent nucleus typical of lymphocyte histology. Importantly, these cells differed morphologically compared to CD14^+^ monocytes (Figure 1, photo 1), which were larger (averaging 15–18 μm in diameter), ameboid in appearance, had a lighter cytoplasm, and unilobar nucleus. This morphological comparison confirms that CD56^neg^CD16^pos^ NK cells are visually indistinguishable from other NK cell subsets and are clearly not monocytes.

### Validating the Purity of Sorted NK Cell Subsets

**Figure 2:**
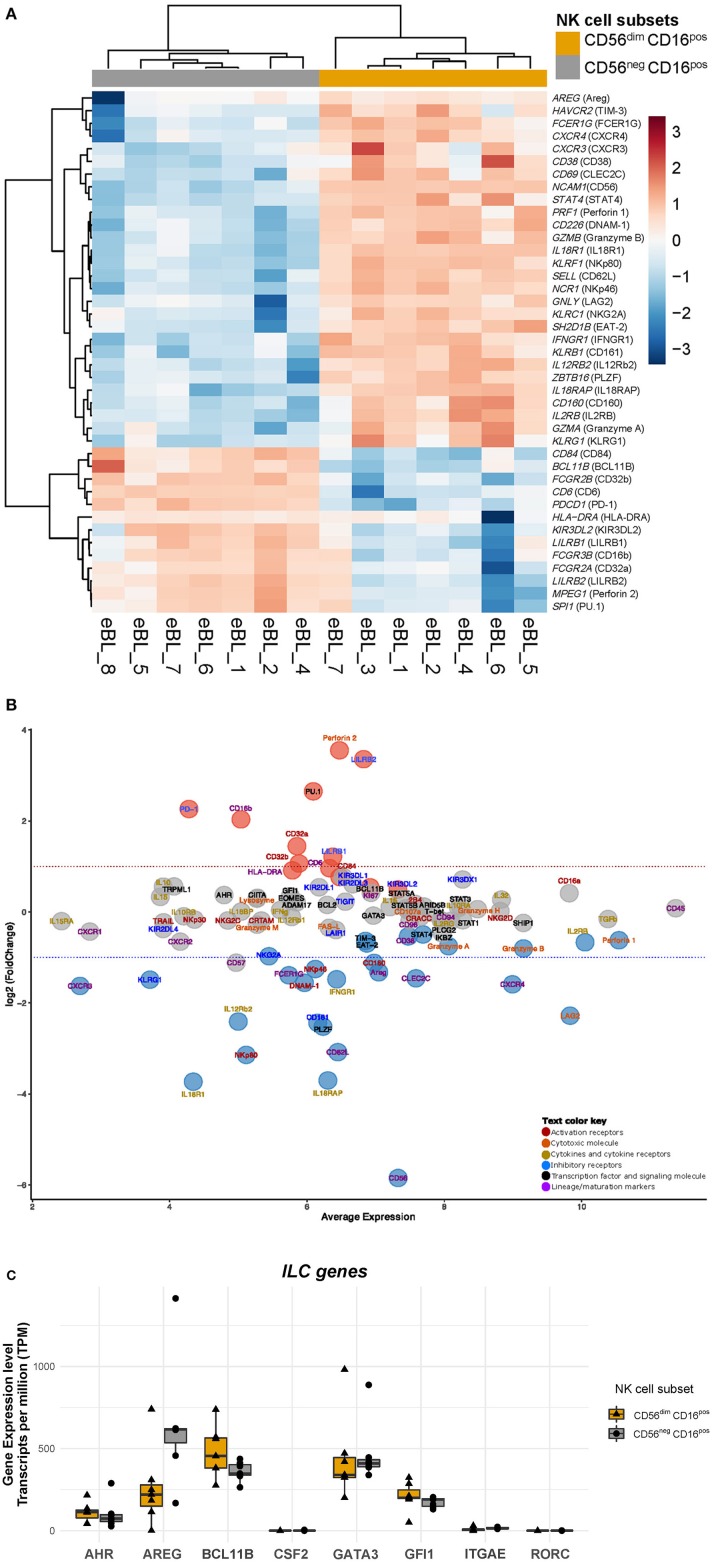
Heatmap and MA plot of selected differentially expressed genes associated with NK cell function. **(A)** Heatmap of selected NK cell genes comparing CD56^neg^CD16^pos^ and CD56^dim^CD16^pos^ NK cells. The id between the brackets are the protein name for that particulate gene, i.e., gene symbol (protein name), NCAM1 (CD56). **(B)** MA plot of differential expression analysis between CD56^neg^CD16^pos^ and CD56^dim^CD16^pos^ NK cell subsets. The MA plot illustrates a log2 fold change of NK specific gene expression in CD56^neg^CD16^pos^ relative to CD56^dim^CD16^pos^ cells and the average normalized expression counts of genes expressed by CD56^neg^CD16^pos^ cells. Protein names are used in the MA plot for ease of comparison to flow data even though these results are for gene expression. The Red dots indicate genes that have significantly higher expression in CD56^neg^CD16^pos^ relative to CD56^dim^CD16^pos^ NK cells (with BH-adjusted *p*-value < 0.01 and FDR < 0.05), whereas blue dots indicate genes that have significantly lower expression (with BH-adjusted *p*-value < 0.01 and FDR < 0.05), and gray dots are genes that are similarly expressed. **(C)** Boxplot of gene expression profiles that define Innate Lymphoid Cells (ILCs): *AHR, AREG, BCL11B, CSF2, GATA3, GFI1, IRGAE, RORC* comparing CD56^neg^CD16^pos^ relative to CD56^dim^CD16^pos^ NK cells.

To check the purity of the sorted CD56^dim^CD16^pos^ and CD56^neg^CD16^pos^ NK cell subsets, we explored the normalized expression values [transcripts per million (TPM)] of each gene, to assess if any of the classical monocyte or neutrophil gene signatures were detected on the bulk RNA-seq data of the sorted cells. From this interrogation, we identified one of the CD56^neg^CD16^pos^ NK samples as having higher expression of classical monocyte genes (CD14 and CD33) (Supplemental Figure 2), suggestive of trace monocyte contamination. Thus, this sample was excluded from further analysis leaving us with 7 CD56^neg^CD16^pos^ and 7 CD56^dim^CD16^pos^ NK subsets for downstream differential expression analysis. All remaining samples lacked expression of CD14, CD33, and CD34 (Supplemental Figure 3A). In addition, we found that neither NK cell subset expressed neutrophil-associated genes, such as CD66b *(CEACAM8), ARG1, MPO, ABCA13, CA1, IFIT1B, CRISP3, LCN2, BPI, CNTNAP3B*, and *PAD4* (Supplemental Figure 3B). However, other neutrophil-associated genes, such as the integrin family genes *ITGAL* (CD11a), *ITGAM* (CD11b), *ITGAD* (CD11d), and *ITGB2* (CD18) which are involved in innate immunity (Roberts et al., 2016) and can be expressed by NK cells were observed in both the CD56^dim^CD16^pos^ and CD56^neg^CD16^pos^ NK cell subsets (Supplemental Figure 3B). In addition, we show that *PTPRC* (CD45) was highly expressed in both the CD56^neg^CD16^pos^ and CD56^dim^CD16^pos^ NK cell subsets (logCPM = 11.37, Figure 2). CD45 is known to be expressed on hematopoietic cells and more highly expressed on lymphoid cells compared to myeloid cells based on BioGPS and MyGene.info organizing online gene-centric information (Wu et al., 2013). This criteria further supports the premise that CD56^neg^CD16^pos^ cells are lymphocytes.

### CD56^neg^CD16^pos^ and CD56^dim^CD16^pos^ NK Cells Transcriptome Expression Profiles

Using purified CD56^dim^CD16^pos^ and CD56^neg^CD16^pos^ NK cell subsets we compared their gene expression profiles and identified 536 genes that were differentially expressed (*p*-value < 0.01 and FDR 0.05) among a combined total of 9,235 genes (Supplemental Figure 4, Supplemental Table 1). Of the differentially expressed genes, 120 had a log Fold Change (logFC) >1.5, with 73 genes downregulated and 47 genes upregulated in CD56^neg^CD16^pos^ relative to CD56^dim^CD16^pos^ NK cells. Each sample showed appropriate expression based on their flow sorting: CD56^dim^CD16^pos^ vs. CD56^neg^CD16^pos^ suggesting minimal cross-contamination. To further characterize the CD56^neg^CD16^pos^ cell subset, we examined the expression of genes that define and distinguish NK cells from other cell types (Figures 2A,B, and Supplemental Table 1). Innate Lymphoid Cells (ILCs) have been categorized into 5 Groups: NK cells that differ based on developmental trajectories from ILC of Group 1 (ILC1) yet both display type 1 immunity, Group 2 (ILC2) able to produce type 2 cytokines, Group 3 (ILC3) defined by their capacity to produce IL-17A and IL-22 and lymphoid tissue-inducer cells (LTi) involved in the creation of secondary lymphoid organ (Vivier et al., 2018). ILC2s are also defined by their expression of *GATA3, BCL11B*, and GFI1 which we found to be expressed at similar levels within CD56^neg^CD16^pos^ relative to the CD56^dim^CD16^pos^ NK cells (Figure 2C). However, amphiregulin (*AREG*) which is expressed by ILC2 group, appeared slightly downregulated in CD56^neg^CD16^pos^ relative to CD56^dim^CD16^pos^ NK cells (logFC of −1.32, *p*-value = 2.24E-06 and FDR of 0.0001). Regarding ILC3 genes, *AHR* was similarly expressed in both NK cell subsets but neither CD56^neg^CD16^pos^ nor CD56^dim^CD16^pos^ cells expressed *CSF2* and *RORC*, considered classical ILC3 genes. Together, the transcriptional profile for CD56^neg^CD16^pos^ cells conform to neither Group 2 nor 3.

### CD56^neg^CD16^pos^ NK Cells Display a Transcriptome Signature Reminiscent of Adaptive NK Cells

Even though 98.7% of genes shared expression between the two NK cell subsets, the differentially expressed genes shed light on functional differences between these two cell populations. Applying our NK-centric transcriptomic analysis, we show that both CD56^neg^CD16^pos^ and CD56^dim^CD16^pos^ NK cells express activation and maturation markers such as *KLRD1* (CD94), CD96, and *B3GAT1* (CD57) (Figure 2B). However, CD56^neg^CD16^pos^ NK cells had lower expression of *CLEC2C* (CD69, logFC of −1.45, *p*-value = 3.96E-10 and FDR of 7.06E-08) and *CD38* (log CPM of 7.47 with logFC of −0.53, *p*-value = 0.0005 and FDR of 0.014) relative to CD56^dim^CD16^pos^ NK cell. In contrast, CD56^neg^CD16^pos^ NK cells tend to express more *HLA-DRA* (logCPM of 5.78 with logFC of 0.91, *p*-value = 0.001 and FDR of 0.03) and *CD6* (logCPM of 6.32 with logFC of 0.96, *p* = 4.84E-06 and FDR of 0.0002) relative to CD56^dim^CD16^pos^ NK cell. As expected, *NCAM-1* (CD56) was strongly downregulated in CD56^neg^CD16^pos^ cells compared to CD56^dim^CD16^pos^ NK cells (logFC of −5.84, *p*-value = 2.17E-198 and FDR of 2.03E-194). Regarding their ability to proliferate, *MKI67* (Ki67) was not differentially expressed (logFC of 0.42, *p*-value = 0.18) between the two subsets. Although, we observed higher expression of *SPI1* (PU.1, logFC of 2.65, *p*-value = 0.0002 and FDR of 0.006) for the CD56^neg^CD16^pos^ relative to CD56^dim^CD16^pos^ NK cells. *SPI1* has been suggested to play a role in NK cell proliferation (Sun, 2016).

Besides potential cytotoxic and pro-inflammatory functions, NK cells have been implicated in regulating immunity by killing activated T cells or antigen-presenting cells (Ferlazzo et al., 2002; Waggoner et al., 2011; Crouse et al., 2014). Therefore, we queried the CD56^neg^CD16^pos^ NK cell transcriptome for immunoregulatory cytotoxic activity. Interestingly, *SELL* (CD62L, logFC of −3.08, *p*-value = 9.28E-17 and FDR of 1.09E-13) and *ZBTB16* (PLZF, logFC of −2.52, *p*-value = 2.65E-14 and FDR of 1.24E-11) gene expression was significantly lower in CD56^neg^CD16^pos^ relative to CD56^dim^CD16^pos^ NK cells, and both *ITGAE* (CD103) and *ITGA*1 (CD49a) were not expressed which suggests that CD56^neg^CD16^pos^ cells may be a form of adaptive NK cell. This is consistent with Schlums et. al who characterized adaptive CD56^dim^ NK cells as CD62L^−^CD103^−^CD49a^−^PLZF1^−^ (Schlums et al., 2015). Moreover, *FCER1G* (FCεRγ) was strongly downregulated (logFC of −1.39, *p*-value = 8.43E-09 and FDR of 1.05E-06, respectively) and to a lesser extent lower levels of *SH2D1B* (EAT-2) (logFC of −0.69, *p-*value = 4.55E-06, FDR of 0.0002) were observed for CD56^neg^CD16^pos^ compared to CD56^dim^CD16^pos^ NK cells. These genes have been correlated with a loss of immunoregulatory cytotoxic activity (Schlums et al., 2015) further supporting the categorization of CD56^neg^CD16^pos^ NK cells as adaptive NK cells.

### CD56^neg^CD16^pos^ NK Cells Express Higher Levels of *PDCD1* and *LILR* Family Inhibitory Receptors Relative to CD56^dim^CD16^pos^ NK Cells

To evaluate the therapeutic potential of these two NK cell subsets, we examined the expression of common NK cell inhibitory molecules (Figure 2B). Most of the KIR members, *TIGIT* and *LAIR1* were similarly expressed by CD56^neg^CD16^pos^ and CD56^dim^CD16^pos^ cells. But interestingly, CD56^neg^CD16^pos^ NK cells expressed significantly lower *KLRG1* (logFC of −1.48) and *KLRB1* (CD161, logFC of −2.43) compared to the CD56^dim^CD16^pos^ NK cells, whereas *KLRC1* (NKG2A, logFC of −0.97) and *HAVCR2* (TIM-3, −0.63) were only slightly downregulated in CD56^neg^CD16^pos^ relative to CD56^dim^CD16^pos^ NK cells. In contrast, *PDCD1* (PD-1, logFC of 2.26) and *LILR* family members, *LILRB1* (logFC of 1.21) and *LILRB2* (logFC of 3.35) were observed to be upregulated within CD56^neg^CD16^pos^ compared to CD56^dim^CD16^pos^ NK cell. However, *PDCD1* was weakly expressed within CD56^neg^CD16^pos^ cells (logCPM of 4) compared to higher expression of *LILRB1* (logCPM of 6). Together these data suggest that CD56^neg^CD16^pos^ NK cells are not exhausted but do overexpress distinct inhibitory receptors that may pose a challenge to overcome with NK-based immune checkpoint inhibitors.

### CD56^neg^CD16^pos^ NK Cells Express Low NKp80 & NKp46 but High CD16, CD32 and Perforin Relative to CD56^dim^CD16^pos^ NK Cells

We previously showed that CD56^neg^CD16^pos^ NK cells were poorly cytotoxic in K562 co-culture assays (Forconi et al., 2018), however at the transcriptional level we observed similar gene expression of some activation (*KLRC2*/NKG2C, *SLAMF7*/CRACC, *TRAIL, CRTAM*/CRTAM), co-stimulation (*CD244*/2B4, *KLRK1*/NKG2D) and natural cytotoxic receptors (*NCR3*/NKp30) for these two subsets (Figure 2B). Yet, CD56^neg^CD16^pos^ NK cells expressed less *KLRF1* that codes for the natural cytotoxic receptor NKp80 (logFC−3.14, *p*-value = 6.02E-16 and FDR of 4.03E-13), *NCR1* (NKp46, logFC −1.25, *p*-value = 1.13E-09 and FDR of 1.63E-07), *CD226* (DNAM-1, logFC −1.55, *p*-value = 1.66E-11 and FDR of 4.39E-09) and *CD160* (logFC −1.11, *p*-value = 1.72E-08 and FDR of 1.87E-06) which might explain in part the loss of cytotoxicity against K562 cells along with the absence of the CD56 adhesion molecule. In contrast, *FCGR3A* (CD16a) is highly expressed on both CD56^neg^CD16^pos^ NK cells (logCPM of 9.81) and CD56^dim^CD16^pos^ NK cells. CD56^neg^CD16^pos^ NK cells expressed higher levels of *FCGR3B* (CD16b) (logFC of 2.03, *p*-value = 0.0005 and FDR of 0.013), *FCGR2A* (CD32A, logFC of 1.4, *p*-value = 3.69E-10 and FDR of 6.78E-08), and *FCGR2B* (CD32B, logFC of 1.06, *p*-value = 7.68E-07 and FDR of 5.25E-05) relative to CD56^dim^CD16^pos^ NK cells with a slightly elevated expression of *CD84* (logCPM of 6.47, logFC of 0.76, *p*-value = 4.07E-05 and FDR of 0.001), respectively. We were surprised to see significantly elevated expression of cytotoxic molecules such as *MPEG1* (perforin 2) for the CD56^neg^CD16^pos^ compared to CD56^dim^CD16^pos^ NK cells (logFC of 3.55, *p*-value = 1.44E-07 and FDR of 1.25E-05). Despite this interesting observation, other genes involved in direct cytotoxicity, such as *LYZ* (lysozyme)*, LAMP-1* (CD107a), *FASLG* (FAS-L), *GZMH* and *GZMM* (granzyme H and M) were not differentially expressed between the CD56^neg^CD16^pos^ and CD56^dim^CD16^pos^ NK cells, supporting their ability to kill target cells by degranulation and through the FAS-L/FAS pathway. CD56^neg^CD16^pos^ NK cells expressed *PRF1* (perforin 1, logCPM of 10.54, logFC of −0.62), *GZMA* (granzyme A, logCPM of 8.06, logFC of −0.75) and *GZMB* (granzyme B, logCPM of 9.15, logFC of −0.8) these markers were slightly lower for CD56^neg^CD16^pos^ compared to CD56^dim^CD16^pos^ NK cells. Another gene related to cytotoxicity *GNLY* (granulysin or LAG2) was significantly downregulated in CD56^neg^CD16^pos^ relative to CD56^dim^CD16^pos^ NK cells (logCPM of 9.83 and logFC of −2.27). Overall, these data suggest that CD56^neg^CD16^pos^ NK cells retain cytotoxic potential, albeit mediated through different mechanisms compared to CD56^dim^CD16^pos^ NK cells.

### CD56^neg^CD16^pos^ NK Cells Express Less IL-2, IL-12, and IL-18 Receptors Relative to CD56^dim^CD16^pos^ NK Cells

As shown in Figure 2B, we found no differences in *IL2RG* gene expression (subunit γ of the IL2 receptor), *IL15RA* (receptor for IL15), *IL12Rb1* (subunit β 1 of the IL12 receptor) between these two NK cell subsets, whereas the other chain of the IL12 receptor (*IL12Rb2*, logFC of −2.4, *p*-value = 7.95E-17 and FDR of 1.06E-13) was found to be significantly downregulated in CD56^neg^CD16^pos^ relative to CD56^dim^CD16^pos^ NK cells. Significant differences were also observed for *IFNGR1* (logFC of −1.48, *p*-value = 2.41E-14 and FDR of 1.19E-11), and both *IL18R1* (IL18 receptor, logFC of −3.73, *p*-value = 4.65E-16 and FDR of 3.35E-13) and *IL18RAP* (IL18 receptor accessory protein, logFC of −3.7, *p*-value = 2E-23 and FDR of 6.25E-20). However, *IL18BP* which encodes the IL18 binding protein is similarly expressed in both CD56^neg^CD16^pos^ and CD56^dim^CD16^pos^ NK cells (logFC of 0.007). Interestingly, *CD122* (IL2RB/IL15RB) appeared to be slightly downregulated in CD56^neg^CD16^pos^ relative to CD56^dim^CD16^pos^ NK cells (logFC of −0.66, *p*-value = 4.85E-05 and FDR of 0.001). These observations suggest that CD56^neg^CD16^pos^ may be impervious to activation by IL2, IL12, IL15, and IL18 cytokines.

### CD56^neg^CD16^pos^ Cells Do Not Differ From CD56^dim^CD16^pos^ NK Cells in Cytokine Gene Expression

We assessed the cytokine expression for CD56^neg^CD16^pos^ and CD56^dim^CD16^pos^ NK cells without any *in vitro* pre-stimulation (Figure 2B). We did not observe any differences in basal expression levels for IL10, IL15, IL16, IL32, or IFNγ between the two NK subsets. However, we observed that both NK cell subsets expressed high TGFβ levels (logCPM of 10.38) which suggests an inherent anti-inflammatory role.

### Validation of Gene Expression by Flow Cytometry

**Figure 3:**
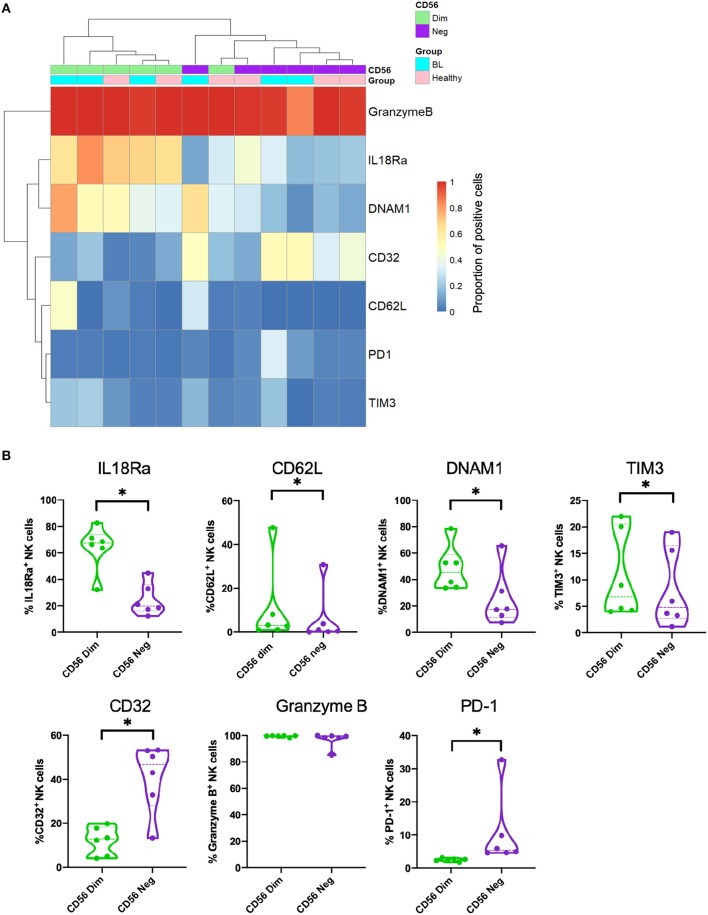
Validation of RNA-sequencing data by flow cytometry. **(A)** Heatmap of markers expressed on both CD56^dim^CD16^pos^ (in green) and CD56^neg^CD16^pos^ (in purple) NK cells from eBL (in turquoise) and healthy (in pink) children. **(B)** Violin plots showing the protein expression of IL18Ra, CD62L, DNAM1, TIM3, CD32, granzyme B and PD-1 from both CD56^dim^CD16^pos^ (in green) and CD56^neg^CD16^pos^ (in purple) NK cells. *Represents a *p*-value < 0.05.

**Figure 4:**
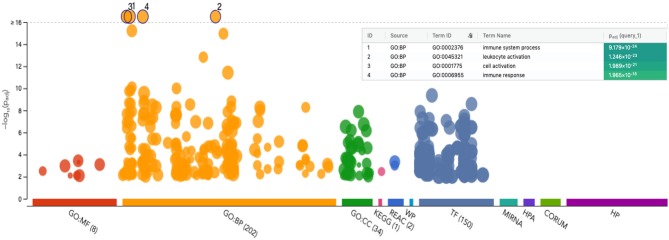
Manhattan plot of functional profiling of the list of upregulated genes from CD56^neg^CD16^pos^ relative to CD56^dim^CD16^pos^ cells. Using the online tool gProfiler and the ordered g:GOSt query, we assessed which biological process (BP) will be linked to the list of 536 significantly differentially expressed genes from CD56^neg^CD16^pos^ relative to CD56^dim^CD16^pos^ cells. The x-axis represents functional terms that are grouped and color-coded by data sources [molecular function (MF), biological process (BP), cell component (CC)]. The y-axis shows the adjusted enrichment *p*-values in negative log10 scale. Adjusted *p*-values g:GOSt used Bonferroni correction and a threshold of 0.01. On the table, adjusted *p*-values were color coded as yellow for insignificant findings to dark blue with highest significance.

Most of the genes differentially expressed when comparing CD56^neg^CD16^pos^ to CD56^dim^CD16^pos^ NK cells that are highlighted in the RNA-sequencing experiment have already been described at the protein expression level in previous studies (Forconi et al., 2018; Voigt et al., 2018). CD56^dim^CD16^pos^ compared to CD56^neg^CD16^pos^ NK cells express less NKp80, IL18Ra, CD161, NKp46, DNAM1 with no differences for CD57, Perforin 1, CD11c, NKG2D, NKG2C and most of the KIRs. We performed additional flow cytometry experiments and confirmed that CD56^dim^CD16^pos^ and CD56^neg^CD16^pos^ NK cells cluster separately (Figure 3A). We also confirmed higher expression of IL18Ra, CD62L, DNAM1, and TIM-3 on the CD56^dim^CD16^pos^ compared to CD56^neg^CD16^pos^ NK cells and higher expression of CD32 and PD-1 on the CD56^neg^CD16^pos^ compared to CD56^dim^CD16^pos^ NK cells (Figure 4B) but no difference in granzyme B expression, thereby validating our RNAseq results.

### Biological Processes Enriched Using gProfiler

Because of the low number of genes differentially expressed between CD56^neg^CD16^pos^ and CD56^dim^CD16^pos^ NK cells, no gene set enrichment was suitable within the GSEA software. However, using the free online tool gProfiler (Figure 4), we were able to visualize which biological processes were enriched using the 536 significantly differentially expressed genes. The Manhattan plot shows the most significant biological processes involved are immune system processes, leukocyte activation, cell activation, and immune response. These results are consistent with our previous observations that CD56^neg^CD16^pos^ NK cells are activated and should be able to drive cytotoxic responses although through alternative pathways to those commonly used by other NK cell subsets.

## Discussion

In this study, we have shown that CD56^neg^CD16^pos^ NK cells share morphological and transcriptional profiles with CD56^dim^CD16^pos^ NK cells. In fact, CD56^neg^CD16^pos^ cells expressed multiple NK cell markers including KIRs, NKG2C, NKp30, CD16a, NKG2D, 2B4, CD57, TRAIL, CRTAM and CRACC. Moreover, our previous study highlighted the phenotypic similarities between these two NK cell subsets (Forconi et al., 2018) which was supported by the Voigt et.al proteomic study (Voigt et al., 2018). Together, these findings confirm that CD56^neg^CD16^pos^ cells are true NK cells. However, we find interesting differences between these two NK cell subsets that might impact functional differences and potential targets that may be potentially harnessed therapeutically to drive NK cell-mediated cytotoxicity.

First, we showed that CD56^neg^CD16^pos^ cells expressed a unique inhibitory marker profile with higher *LILR* family and *PDCD1* gene expression. *LILRB1* encodes for LILRB1 transmembrane receptors which contain 4 ITIMs motif in the cytoplasmic tail and is expressed by various immune cells (Zhang et al., 2015). After stimulation by its ligands, various HLA class I molecules among others, trigger a strong inhibition signal in order to limit inflammatory and cytotoxic responses. Its most efficient ligand is a dimerized HLA-G which was described as up-regulated in some human tumors such as breast cancer (Lefebvre et al., 2002), certain AML (acute myeloid leukemia) (Kang et al., 2015) and cutaneous T cell lymphoma (Urosevic et al., 2004) in which both CD8^+^ T and CD56^+^ NKT cells highly expressed LILRB1 and thereby possibly contributed to tumor immune escape. More recently, LILRB1 blockade was shown to enhance cytotoxic CD8^+^ T cell activity using bispecific T cell engager (BiTE) (Kim et al., 2019) molecules, highlighting the potential of the LILRB1 receptor as an anti-cancer therapeutic target. LILRs are not known to be included in a T cell exhaustion signature (McLane et al., 2019), contrary to other markers such as PD-1, TIM-3, TIGIT, LAG3, CTLA-4, KLRG1, BTLA, CD160, and 2B4. In our study, *PDCD1*, coding for the PD-1 protein, was more highly expressed for CD56^neg^CD16^pos^ relative to CD56^dim^CD16^pos^ NK cells even though its expression was very low compared to most other genes of interest (Figure 2B). At the proteome level, PD-1 was not differentially expressed across NK cell subsets (Voigt et al., 2018). PD-1 is a popular target for immune checkpoint inhibitors, although insufficient in isolation to determine the extent it plays in T cell exhaustion (Blank et al., 2019; McLane et al., 2019). In this viewpoint article, the authors suggested the potential for adaptation of T cells into an exhaustion profile in order to limit immunopathology during chronic infections (Blank et al., 2019). Similarly, we suggest that CD56^neg^CD16^pos^ NK cells are an adaptation of CD56^dim^CD16^pos^ NK cells under conditions of chronic infections or persistent tumor ligand stimulation. In our study of eBL patients, we observed that KLRG1, TIM-3 and CD160 had lower expression in CD56^neg^CD16^pos^ relative to CD56^dim^CD16^pos^ NK cells. Moreover, CTLA-4 and BTLA were not expressed at all, which suggests that the CD56^neg^CD16^pos^ NK subset does not appear to have a more exhausted profile than the other NK cells subsets but they clearly express multiple inhibitory markers which could limit immune responses.

As NK cells function depend on multiple signals triggered by both inhibitory and activation receptors, we assess as well which activation and cytotoxic markers were strongly differentially expressed between CD56^dim^CD16^pos^ and CD56^neg^CD16^pos^ NK cells. Despite numerous markers similarly expressed (CD6, HLA-DR, CD57, CD84, TRAIL, NKp30, NKG2C, NKG2D, 2B4…), CD56^neg^CD16^pos^ NK cells showed a strong downregulation of *KLRF1* coding for the cytotoxic receptor NKp80 and to a lesser extent *NCR1* coding for NKp46 relative to CD56^dim^CD16^pos^ NK cells. A recent study described NKp80 as a marker of NK cell maturity (Freud et al., 2016). In brief, they characterized NKp80^neg^ NK cells from secondary lymphoid tissues as stage 4a of NK cell development, which also included low expression of perforin, T-bet, EOMES, lack of Granzymes A, B and K but higher expression of *RORC2* and *AHR* (features shared with ILC3). Despite the expression of *AHR* in both CD56^dim^CD16^pos^ and CD56^neg^CD16^pos^ NK cell subsets, *RORC2* was absent and we didn't observe a significant differential expression of T-bet and EOMES genes. However, Perforin 1, granzyme A and B genes appeared to be slightly downregulated within the CD56^neg^CD16^pos^ NK cells. Regarding NKp46, we have previously shown that the expression of this cytotoxic receptor is significantly lower for children exposed to *Plasmodium falciparum* (Forconi et al., 2018). These data suggest another role for NKp80 and potentially an adaptation of NKp46 expression within eBL children within the context of malaria, EBV co-infections and the eBL pathogenesis. Despite the less expression of cytotoxic receptors essential in natural direct cytotoxicity, NK cells have other ways to kill target cells involving the presence of IgG antibodies. Interestingly, we observed upregulation of low-affinity Fcγ receptor CD16, the medium-affinity Fcγ receptor CD32 and Perforin 2 genes within the CD56^neg^CD16^pos^ compared to the CD56^dim^CD16^pos^ NK cell subset. There are two genes which encode the CD16 protein: *FCGR3A* and *FCGR3B* and they share more than 95% of homology so that common CD16 flow cytometry antibodies cannot distinguish them from one another (Ravetch and Perussia, 1989). However, the expression of these two genes were described as cells-specific and with different functions from CD16b which is a glycophosphatidylinositol (GPI) -anchored molecule without intracellular signaling motifs. In order to measure cells specificity of both CD16a and CD16b, Ravetch's team reconstituted these receptors in transgenic mice (Li et al., 1996). They showed that CD16a is expressed by macrophages and NK cells whereas CD16b is exclusively expressed by neutrophils. In our study we show that CD16a is highly and similarly expressed by CD56^dim^CD16^pos^ and CD56^neg^CD16^pos^ NK cells, however, CD16b is significantly upregulated within CD56^neg^CD16^pos^ NK cell subset. CD16a or *FCGR3A* receptor is known as an important mediator of antibody dependent cell cytotoxicity (ADCC), an indirect mechanism used by NK cells and other innate immune cells to kill tumors and infected host cells (Hart et al., 2017; Arora et al., 2018; Victor et al., 2018). CD16b has been implicated as an essential mediator of antibody-dependent respiratory burst (ADRB) by neutrophils and has also been shown to be essential for immune complexes (ICs) but not necessarily involved in phagocytosis (Fossati et al., 2002). A recent study showed that CD16b can also regulate ADCC by neutrophils in competition with CD16a (Treffers et al., 2018), and in fact low copy number variation (CNV) of the gene *FCG3RB* within activated cells might increase ADCC capacity. Our transcriptome analysis was not able to assess CNV although we observed a logCPM of 5.03 for CD16b which is twice less than logCPM of 9.81 for CD16a. It will be important to consider CNV in future experiments in order to clearly determine the role of CD16b within NK cells. Both *FCGR2A* (CD32a) and *FCGR2B* (CD32b) were also more highly expressed by CD56^neg^CD16^pos^ than CD56^dim^CD16^pos^ NK cells. These findings suggest that CD56^neg^CD16^pos^ NK cells may be superior in recognizing antibody opsonized targets. Using recognition of the complement, *CD11b* and *CD18* were found expressed within NK cells from our study population. The heterodimer CD11b/CD18 is called complement receptor 3 (CR3, Mac1 or α_M_β_2_). CR3 is a multi-functional receptor which was described as predominantly expressed on myeloid and NK cells (Ross and Vetvicka, 1993; Vorup-Jensen and Jensen, 2018) and involved in NK cell cytotoxicity (Lee et al., 2017). CR3 can interact with Fc receptors for adhesion to immune complexes and therefore enhance cell mediated antibody-dependent cytotoxicity (Ortiz-Stern and Rosales, 2003).

Cytokines also play an essential role in NK cell activation and function. In this study we observed a strong significant downregulation of IL18 receptor (*IL18RAP* and *IL18R1*) as well as IL12RB2, β chain of IL12 receptor within CD56^neg^CD16^pos^ relative to CD56^dim^CD16^pos^ NK cells. IL18 and IL12 are both known to be crucial in NK cell activation, therefore, the downregulation of these receptors on CD56^neg^CD16^pos^ NK cells might impair their activation. However, it was previously shown that both IL18 and IL12 receptors are silenced in adaptive CD56^dim^ NK cells in order to block their ability to produce immunoregulatory cytokines (Schlums et al., 2015), whereas the IL15 receptor (*IL15RA*) is still expressed. We also show that CD56^neg^CD16^pos^ NK cells are CD62L^−^CD103^−^CD49a^−^PLZF1^−^ with a downregulation of *FCER1G* (FCεRγ) and *SH2D1B* (EAT-2). This phenotype has been correlated with a loss of immunoregulatory cytotoxic activity (Schlums et al., 2015). Similar observations were made for HIV-positive individuals who had broadly reactive neutralizing antibodies (bnAbs), lower expression of IL12 and IL18 receptors and PLZF1 yet with higher expression of CD6 (Bradley et al., 2018), confirming an adaptive-like NK cell phenotype. In addition, HIV-positive individuals with bnABs had a higher proportion of CD56^neg^CD16^pos^ NK, which was associated with better viral control. Interestingly, a positive correlation was observed between CD56^neg^CD16^pos^ NK cells and mRNA expression of *RAB11F1P5*, encoding for Rab11 recycling endosome molecule, which was also found to be increased in CD56^neg^CD16^pos^ NK cells from children in our study. Together, these studies support the development of adaptive CD56^neg^CD16^pos^ NK cells as adaptation to chronic infections.

**Figure 5:**
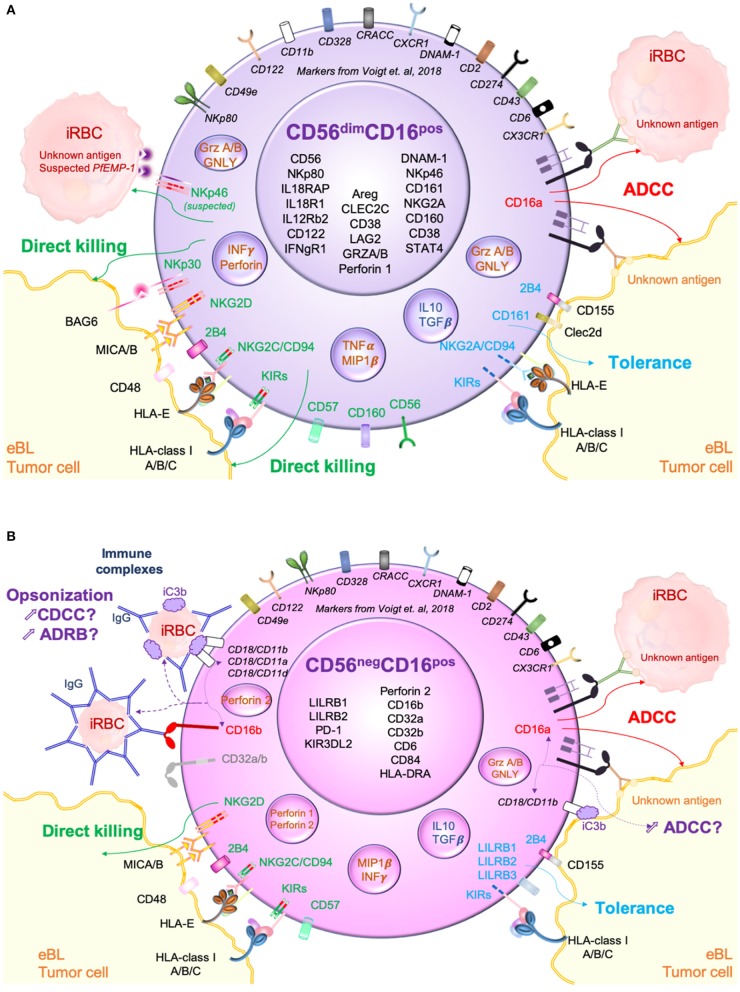
Hypothetical model of CD56^dim^CD16^pos^ and CD56^neg^CD16^pos^ NK cells cytotoxic pathways against both *Plasmodium falciparum*-infected red blood cells (*Pf*-iRBC) and endemic Burkitt lymphoma (eBL) tumor cells. **(A)** Proposed killing pathways used by CD56^dim^CD16^pos^ NK cells against iRBCs and eBL tumors primarily mediated by natural direct cytotoxicity (direct killing) through the activation of cytotoxic receptors (NKp46, NKp30, NKp80, NKG2D…) but also antibody dependent cell cytotoxicity (ADCC) through CD16a activation. **(B)** Proposed killing pathways used by CD56^neg^CD16^pos^ NK cells against iRBC and eBL tumors. Due to the low expression of cytotoxic receptors, direct killing appears to be incapacitated however other killing pathways based on recognition of opsonized targets might be enhanced.

In summary, we present a hypothetical model (Figure 5) contrasting killing pathways used by CD56^dim^CD16^pos^ and CD56^neg^CD16^pos^ NK cells against eBL tumor cells as well as *P. falciparum*-infected red blood cells (iRBC). *P. falciparum* has been consistently linked to eBL pathogenesis (Moormann and Bailey, 2016). Here, we hypothesize that continual malaria infections induce NK cell adaptation thereby increasing the prevalence of CD56^neg^CD16^pos^ NK cells. Figure 5A illustrates how CD56^dim^CD16^pos^ NK cells kill iRBC after direct recognition between NKp46 and its putative ligand, *P. falciparum* erythrocyte membrane protein 1 (*Pf* EMP-1) (Wolf et al., 2017). CD56^dim^CD16^pos^ NK cells also use direct recognition to kill tumor cells by activation of multiple receptors. However, KIRs, NKG2A/CD94 and 2B4 can trigger tolerance and therefore allow immune escape of the tumor cells. Determining which NK cell inhibitory ligands are expressed by tumors is an area of ongoing investigation. Finally, in the presence of antibodies directed against iRBCs and tumor cells neo-antigens, CD56^dim^CD16^pos^ NK cells can kill both tumor cells and iRBCs through ADCC triggered by CD16a. ADCC against iRBCs has already been well-described by others (Hart et al., 2017; Arora et al., 2018). In contrast, we hypothesize that the main method of killing for CD56^neg^CD16^pos^ NK cells is through antibody dependent cytotoxicity (Figure 5B). Expression of all CD18/CD11_a/b/c_ as well as CD16a, CD16b, CD32a, CD32b and Perforin 2 suggests a multifaceted involvement of antibody opsonization in the killing capacity of CD56^neg^CD16^pos^ NK cells. *P. falciparum* induces a broad range of antibodies directed against the many parasite antigens that are able to trigger ADCC and ADRB (Moormann et al., 2019). Another important component for killing target cells is complement. Present in plasma, iC3b can be fixed on iRBCs as well as tumor cells and thereby opsonize the target for innate cells. This immune complex can be recognized by CR3 expressed on NK cells and trigger complement dependent cell-mediated cytotoxicity (CDCC) against the target cells. Moreover, it was shown that CR3 should be able to communicate with the Fcγ receptors (CD16a/b) and therefore be able to enhance ADCC and ADRB functions (Lee et al., 2017). We therefore suspect that ADCC can as well be important against tumor cells if the tumor is expressing surface antigens for antibody opsonization. In contrast, we and others have shown that CD56^neg^CD16^pos^ NK cells are not well-adapted for natural direct cytotoxicity because of their strong downregulation of multiple cytotoxic and activation receptors. Thus, CD56^neg^CD16^pos^ NK cell abundance appears to be a refined adaptation influenced by chronic diseases, that focuses NK cell mediated cytotoxicity toward antibody opsonized targets.

As a limitation of our study, bulk RNA-sequencing didn't allow us to assess the copy number variation (CNV) that can impact the role of CD16b on ADCC. CD56^neg^CD16^pos^ NK cells might use other means to kill target cells such as ADRB or CDCC. These pathways will need to be assessed in functional assay in order to determine which cytotoxic mechanisms are engaged by CD56^neg^CD16^pos^ NK cells.

## Data Availability Statement

The datasets generated for this study can be found in the NCBI's database of Genotypes and Phenotypes (dbGaP) with accession number phs1282.V2.

## Ethics Statement

The studies involving human participants were reviewed and approved by University of Massachusetts Medical School Institutional Review Board and the Scientific and Ethical Review Unit at the Kenya Medical Research Institute. Written informed consent to participate in this study was provided by the participants' legal guardian/next of kin.

## Author Contributions

CF designed research, performed research, analyzed data, and wrote the paper. CO designed research, performed research, analyzed data, and reviewed the paper. PO performed research and reviewed the paper. JO contributed to samples acquisition and reviewed the paper. CM reviewed the paper. JB contributed experimental tools, analyzed data, and reviewed the paper. AM designed research, contributed experimental tools, analyzed data, and reviewed the paper.

## Conflict of Interest

The authors declare that the research was conducted in the absence of any commercial or financial relationships that could be construed as a potential conflict of interest.

## Supplementary Material

Supplemental Table 1 Genes significantly differentially expressed between the CD56^neg^CD16^pos^ NK cells and CD56^dim^CD16^pos^ NK cells (Benjamini-Hochberg procedure (BH) adjusted *p*-value < 0.01 and false discovery rate (FDR) < 0.05).

Supplemental Figure 1 CD56^neg^CD16^pos^ NK cells across population. **(A)** Representative cytoplots of NK cell subsets repartition from PBMCs within healthy American and Kenyan adults as well as healthy and eBL Kenyan children. CD56^pos^ NK cells are gated in red box and CD56^neg^ in blue box. **(B)** Percentage of CD56^neg^CD16^pos^ NK cells across our different groups: healthy American, healthy Kenyan, healthy children from Nandi (EBV+/*Pf*-), healthy children from Kisumu (EBV+/*Pf* +) and eBL children (EBV+/*Pf* +). ^****^Represents a *p*-value ≤ 0.0001; ^***^represents a *p*-value ≤ 0.001; ^**^represents a p-value ≤ 0.01. ^*^Represents a *p*-value < 0.05.

Supplemental Figure 2 Excluding NK subsets with possible monocyte contamination. Boxplot showing the expression of monocyte associated genes (*CD14, CD33*, and *CD34*). We identified one of the sorted CD56^dim^CD16^pos^ NK cells (highlighted red in the boxplot), to have elevated expression of monocyte marker genes (CD14 and CD33). Elevated expression of these 2 genes could have been due to monocyte contamination during the sorting process of that particular sample. This sample was excluded from all downstream analysis.

Supplemental Figure 3 Purity of flow sorted CD56^neg^CD16^pos^ and CD56^dim^CD16^pos^ cell subsets. **(A)** Boxplot of monocyte gene expression profile *CD14, CD33*, and *CD34* genes expression within both CD56^neg^CD16^pos^ and CD56^dim^CD16^pos^ NK cells. **(B)** Boxplot of neutrophil gene expression profile *ARG1, ARHGEF12, BPI, CA1, CEACAM8, CNTNAP3B, CRISP3, FUT4, IFIT1B, ITGAM, ITGB2, LCN2*, and *MPO* genes expression within both CD56^neg^CD16^pos^ and CD56^dim^CD16^pos^ NK cells. ^***^Represents a *p*-value ≤ 0.00001; ^*^represents a *p*-value ≤ 0.01.

Supplemental Figure 4 Heatmap of all differentially expressed genes for CD56^neg^CD16^pos^ compared to CD56^dim^CD16^pos^ NK cells. Differential gene expression analysis identified 536 genes to be significantly differentially expressed (Benjamini-Hochberg method [BH] adjusted *p*-value < 0.01 and False Discovery Rate (FDR) < 0.05) between the CD56^neg^CD16^pos^ and the CD56^dim^CD16^pos^ NK cell subsets. The heatmap shows hierarchical clustering based on the expression profile of the 536 differentially expressed genes between the two NK cell subsets. The color key indicates the intensity associated with normalized expression values. Of the 536 genes identified to be differentially expressed between these two NK cell subsets, 350 genes were downregulated among the CD56^neg^CD16^pos^ cells compared to the CD56^dim^CD16^pos^ and 186 genes were upregulated in the CD56^neg^CD16^pos^ cells compared to the CD56^dim^CD16^pos^ cells (Supplemental Table 1).
