# Supplementary figures and images for: A New Hope for CD56negCD16pos NK Cells as Unconventional Cytotoxic Mediators: An Adaptation to Chronic Diseases
Source: Front Cell Infect Microbiol. 2020 Apr 21;10:162. doi: 10.3389/fcimb.2020.00162 (PMC7186373; doi:10.3389/fcimb.2020.00162)

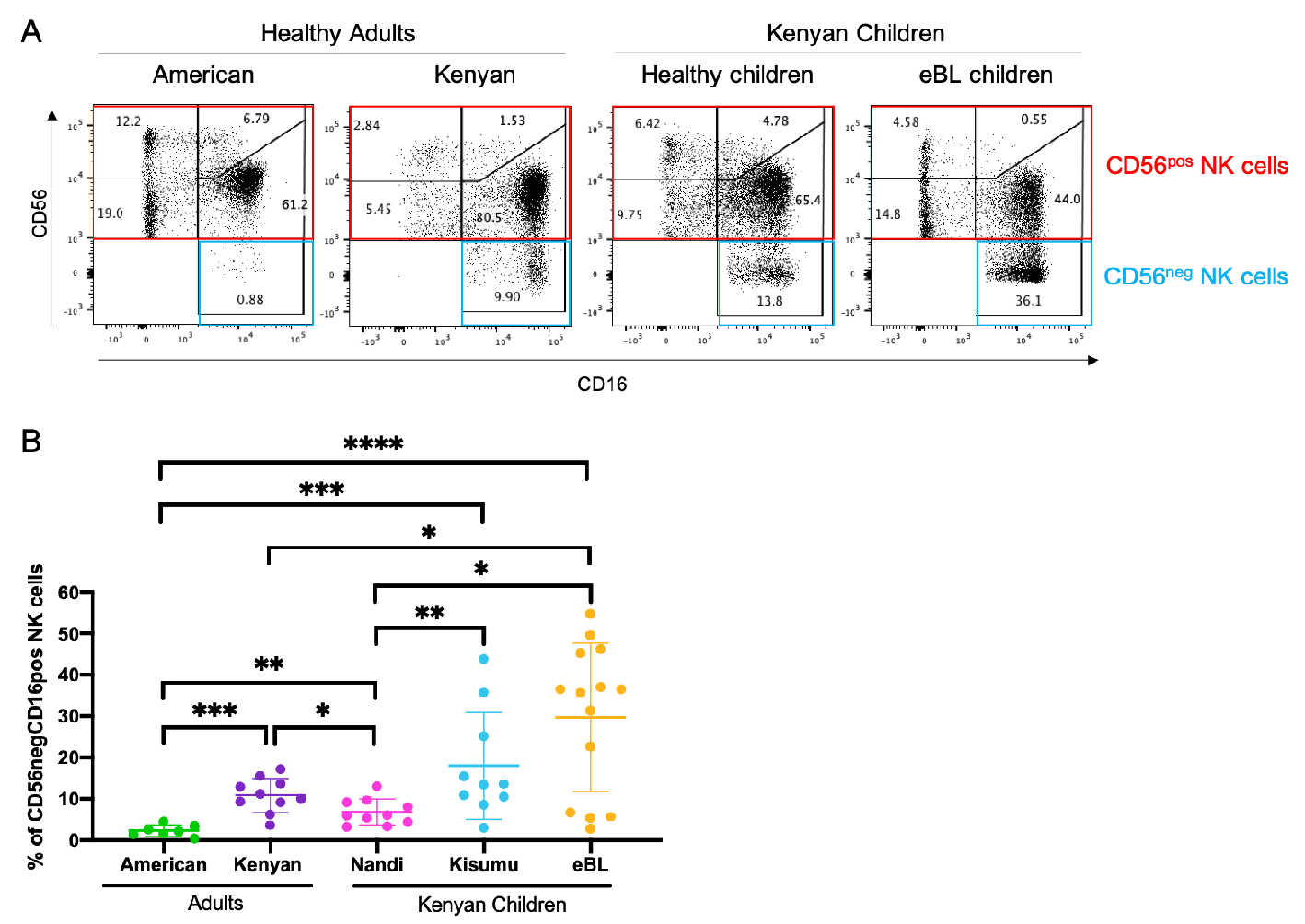

Supplement: Supplemental Figure 1 — CD56negCD16pos NK cells across population. (A) Representative cytoplots of NK cell subsets repartition from PBMCs within healthy American and Kenyan adults as well as healthy and eBL Kenyan children. CD56pos NK cells are gated in red box and CD56neg in blue box. (B) Percentage of CD56negCD16pos NK cells across our different groups: healthy American, healthy Kenyan, healthy children from Nandi (EBV+/Pf-), healthy children from Kisumu (EBV+/Pf +) and eBL children (EBV+/Pf +). ****Represents a p-value ≤ 0.0001; ***represents a p-value ≤ 0.001; **represents a p-value ≤ 0.01. *Represents a p-value < 0.05. [file Image_1.TIF]

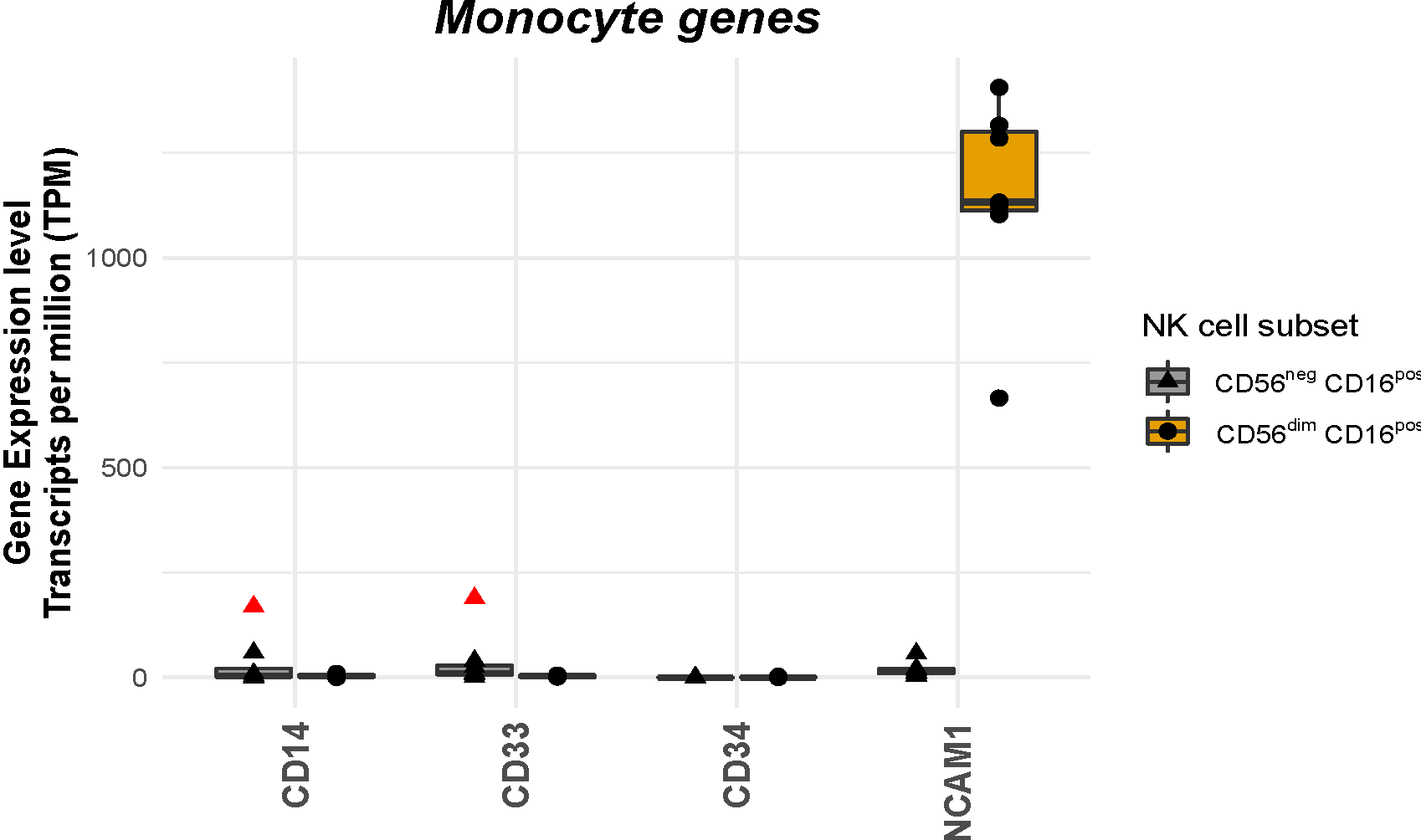

Supplement: Supplemental Figure 2 — Excluding NK subsets with possible monocyte contamination. Boxplot showing the expression of monocyte associated genes (CD14, CD33, and CD34). We identified one of the sorted CD56dimCD16pos NK cells (highlighted red in the boxplot), to have elevated expression of monocyte marker genes (CD14 and CD33). Elevated expression of these 2 genes could have been due to monocyte contamination during the sorting process of that particular sample. This sample was excluded from all downstream analysis. [file Image_2.TIF]

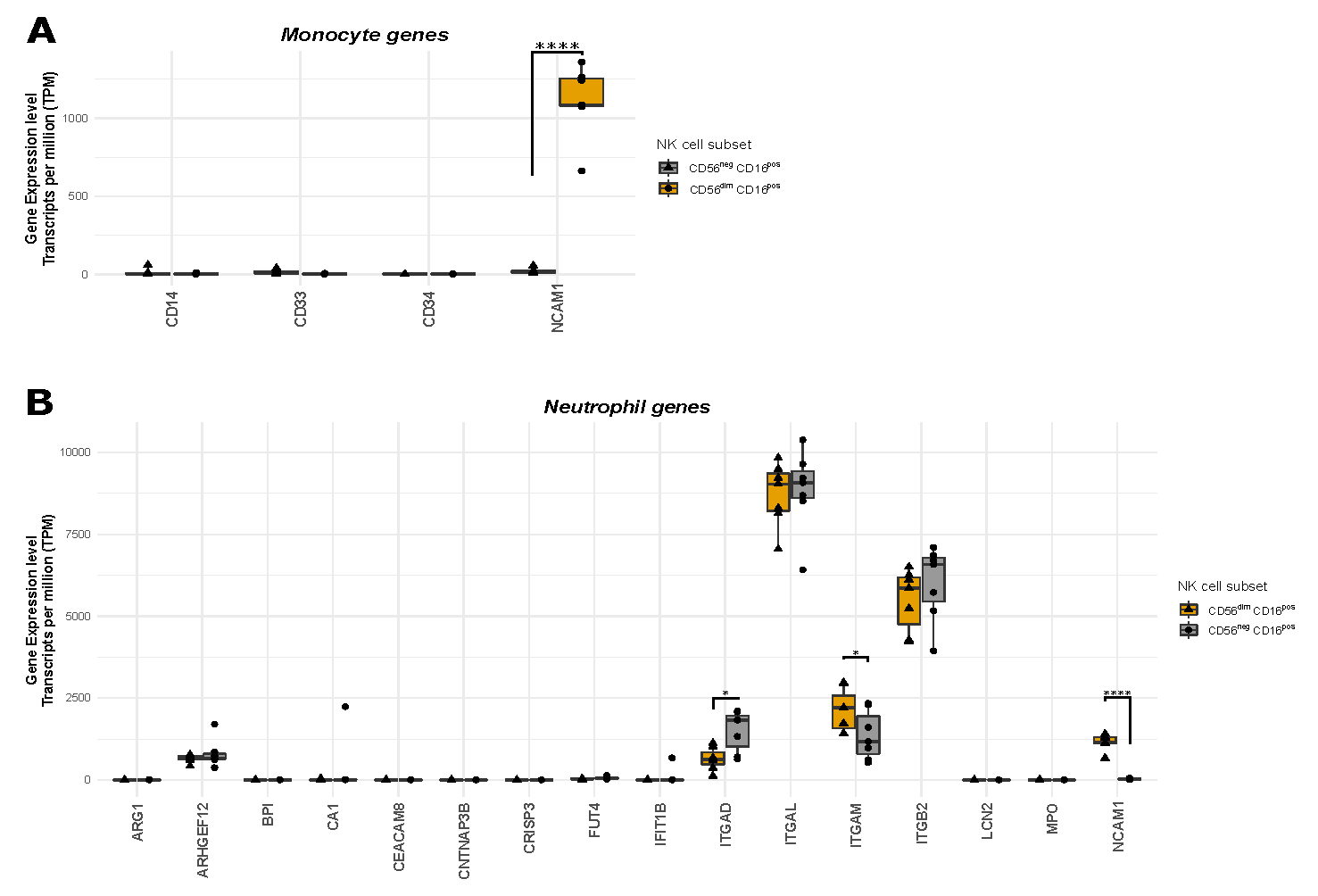

Supplement: Supplemental Figure 3 — Purity of flow sorted CD56negCD16pos and CD56dimCD16pos cell subsets. (A) Boxplot of monocyte gene expression profile CD14, CD33, and CD34 genes expression within both CD56negCD16pos and CD56dimCD16pos NK cells. (B) Boxplot of neutrophil gene expression profile ARG1, ARHGEF12, BPI, CA1, CEACAM8, CNTNAP3B, CRISP3, FUT4, IFIT1B, ITGAM, ITGB2, LCN2, and MPO genes expression within both CD56negCD16pos and CD56dimCD16pos NK cells. ***Represents a p-value ≤ 0.00001; *represents a p-value ≤ 0.01. [file Image_3.TIF]

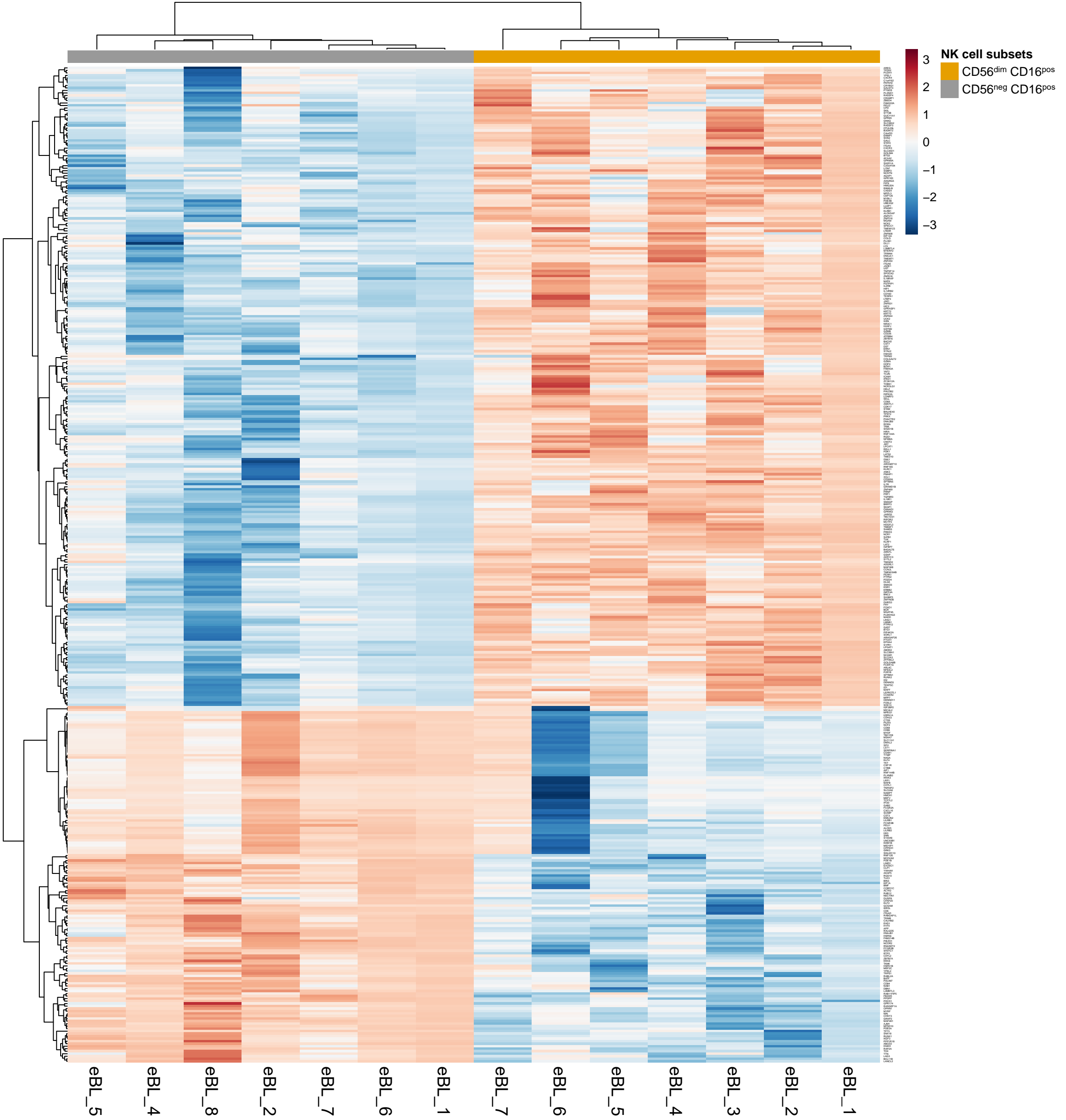

Supplement: Supplemental Figure 4 — Heatmap of all differentially expressed genes for CD56negCD16pos compared to CD56dimCD16pos NK cells. Differential gene expression analysis identified 536 genes to be significantly differentially expressed (Benjamini-Hochberg method [BH] adjusted p-value < 0.01 and False Discovery Rate (FDR) < 0.05) between the CD56negCD16pos and the CD56dimCD16pos NK cell subsets. The heatmap shows hierarchical clustering based on the expression profile of the 536 differentially expressed genes between the two NK cell subsets. The color key indicates the intensity associated with normalized expression values. Of the 536 genes identified to be differentially expressed between these two NK cell subsets, 350 genes were downregulated among the CD56negCD16pos cells compared to the CD56dimCD16pos and 186 genes were upregulated in the CD56negCD16pos cells compared to the CD56dimCD16pos cells (Supplemental Table 1). [file Data_Sheet_1.PDF]
